# Differential expression of genes associated with lipid import, β-oxidation and lactate oxidation induced by Mycobacterium tuberculosis curli pili in broth culture compared to intracellular bacilli within THP-1 macrophages

**DOI:** 10.1099/jmm.0.001994

**Published:** 2025-03-31

**Authors:** Shinese Ashokcoomar, Manormoney Pillay

**Affiliations:** 1 Medical Microbiology, School of Laboratory Medicine and Medical Sciences, College of Health Sciences, University of KwaZulu-Natal, 1st floor Doris Duke Medical Research Institute, Congella, Private Bag 7, Durban 4013, South Africa

**Keywords:** adhesin, gene expression, *Mycobacterium tuberculosis*, reverse transcription real-time quantitative PCR, THP-1 macrophage infection

## Abstract

**Introduction.** The adhesin, *Mycobacterium tuberculosis* curli pili (MTP), assists the pathogen in attachment, invasion and disease progression. Previously, this adhesin was demonstrated to contribute to the pathogen’s cell wall functions and fatty acid metabolism and affects total metabolite abundance in central carbon metabolism and fatty acid metabolism of the host. The accumulation/depletion of metabolites is reliant on the gene expression of proteins involved in the import, transport and breakdown of substrates.

**Gap statement.**MTP has not been investigated in relation to genes involved in import/transport/breakdown of substrates.

**Aim.** This study aimed to investigate the possible regulatory role of MTP in modulating metabolic changes of the pathogen in different microenvironments.

**Methods.** Ribonucleic acid was harvested from bacterial broth cultures of adhesin-proficient and adhesin-deficient *M. tuberculosis*. These strains were also used to infect differentiated THP-1 macrophages for 4 h prior to isolation of intracellular bacteria, RNA extraction and reverse transcription real-time quantitative PCR. The expression levels of selected genes involved in fatty acid transport (*lucA*, *mce1D*, *mceG*, *Rv2799*, *Rv0966c* and *omamB*), β-oxidation (*fadA5* and *fadB*), lactate oxidation (*lldD1* and *lldD2*) and gluconeogenic carbon flow (*pckA*) were analysed by absolute quantification.

**Results.** The gene expression levels of *lucA*, *mce1D* and *pckA* were significantly lower, and those of *Rv2799*, *Rv0966c*, *mceG*, *fadA5* and *lldD2* were significantly higher in the adhesin-proficient cultured bacterial strains relative to the Δ*mtp* strain. The intracellular adhesin-proficient bacteria displayed significantly higher gene expression levels of *Rv2799* and significantly lower gene expression levels of *Rv0966c*, *fadA5*, *lldD1* and *pckA* relative to the Δ*mtp* strain. Interestingly, during early infection, the intracellular Δ*mtp* displayed significantly increased expression of *omamB*, *mceG*, *fadB*, *lldD1* and *lldD2* relative to the broth culture. This trend was inverted in the WT models.

**Conclusion.**MTP are significantly associated with the regulation of genes involved in lipid transport, β-oxidation and lactate oxidation.

## Introduction

Despite the advancements made in the vaccination, diagnosis and treatment of tuberculosis (TB) over the last 142 years, the longstanding battle to eradicate *Mycobacterium tuberculosis *continues. Of the 10.8 million people who fell ill with TB in 2023, 55% were adult males, 33% were adult females and 12% were children [1]. Drug resistance to first-line rifampicin (RR-TB) and isoniazid, known as multidrug-resistant TB (MDR-TB), requires treatment with second-line drugs. In 2023, 3.7% of incident cases were estimated to be MDR/RR-TB, of which only 44% were diagnosed and treated [1]. Hence, intensified research and innovation are urgently required to help meet The End TB Strategy targets set for 2030 and 2035 [1].

The lack of suitable and accurate biomarkers impedes the development of rapid and effective diagnostics and therapeutics that are essential to address the burden of TB. Essential to microbial pathogenesis, extracellular surface molecules/appendages known as adhesins are considered valuable biomarkers [2]. Adhesins facilitate the initial interaction between pathogen and host, subsequently permitting the invasion of the host during infections. *Mycobacterium tuberculosis* curli pili (MTP) has been evidenced as a prominent adhesin of *M. tuberculosis*. This adhesin is encoded by the open reading frame (ORF) *Rv3312A* (*mtp*) and is not arranged as part of an operon or cluster with curli/pili-associated genes [3]. Instead, *mtp* is located between intermediary metabolism-associated genes [4], is highly conserved amongst clinical isolates and is unique to *M. tuberculosis* complex strains [5]. Numerous genomics/functional genomics [5,8], transcriptomics [9,12], biomarker evaluation [5,13] and functional metabolomic studies [14,16] have provided evidence that MTP is a potentially suitable target as a TB diagnostic and vaccine candidate.

As a result of the metabolic alterations observed in the MTP-deficient strain of *M. tuberculosis*, as well as its infected host during early stages of infection, it was proposed that MTP influences the production of metabolites associated with cell composition, carbon metabolism, fatty acid metabolism and amino acid metabolism [14,17]. These metabolic changes are a result of perturbations in gene regulation of various membrane-bound enzymes or genes involved in β-oxidation, lipid transport/metabolism and gluconeogenic flow [14,15]. *M. tuberculosis* can utilize multiple nutrient sources such as sugars, fats and other available carbon-rich compounds [18,20]; hence, an understanding of the function and regulation of the array of genes involved in nutrient assimilation may further elucidate the mechanisms of TB pathogenesis.

The uptake and transport of fatty acids and cholesterol during *M. tuberculosis* infection is mediated by mammalian cell entry (Mce) proteins [21]. Additionally, the genome of *M. tuberculosis* encompasses four unlinked *mce* loci (*mce1–mce4* operons), which are responsible for the transport of lipids into *M. tuberculosis* [22]. The recently identified *Rv2799*, *Rv0966c*, *Rv0200/omamB*, *Rv0172/mce1D* and *Rv0655/mceG* were shown to be involved in fatty acid import and the putative components of the Mce fatty acid transporters [23]. Moreover, lipid uptake coordinator A (*Rv3723/lucA*) was demonstrated to play an important role in *M. tuberculosis* virulence as it facilitates the import of both fatty acids and cholesterol [22,23].

Once transported into the cell, fatty acids and cholesterol need to be broken down before they can be incorporated into the tricarboxylic acid cycle and repurposed within the cell. There are over 100 genes involved in the β-oxidation of fatty acids to produce acetyl-CoA in *M. tuberculosis*, many of which are annotated as non-essential *in vitro*, possibly due to extensive gene redundancy [24]. The multi-catalytic enzyme, FadB, catalyses both the hydration and the 3-hydroxy dehydrogenation steps in the β-oxidation cycle [25]. The FadB protein forms a trifunctional complex with the thiolase FadA (FadA–FadB complex), which mediates three of the four reaction steps in β-oxidation [26]. The β-oxidation of cholesterol is catalysed by acetyl-CoA acetyltransferase (FadA5), which is responsible for thiolysis of acetoacetyl CoA [27].

Intracellular growth of *M. tuberculosis* is brought about by biomass formation through gluconeogenesis. The gluconeogenic carbon flow is mediated by phosphoenolpyruvate carboxykinase (PEPCK), encoded by *pckA* (*Rv0211*), and is considered crucial for the survival of *M. tuberculosis* in macrophages and in mice [28]. Moreover, given that macrophages respond to infection by switching from pyruvate oxidation to pyruvate reduction, lactate is plentiful at the site of infection and is exploited as a carbon substrate by *M. tuberculosis* [29]. In *M. tuberculosis*, the conversion of lactate is strictly dependent on one of two potential l-lactate dehydrogenases. These enzymes are encoded by *lldD1* (*Rv0694*) and *lldD2* (*Rv1872c*) and were demonstrated to play a crucial role in lactate oxidation of this intracellular pathogen [30].

The regulation of the above-mentioned genes could have potentially altered the metabolic profiles reported previously in cultured MTP-deficient *M. tuberculosis*, which displayed an accumulation of fatty acids, cell wall constituents and carbon metabolism intermediates, relative to WT *M. tuberculosis* [15]. Moreover, the MTP-deficient *M. tuberculosis-*infected THP-1 macrophages had higher concentrations of lactate, glyceric acid and fatty acids relative to the WT infected macrophages [14]. Hence, it was postulated that MTP plays a significant role in the regulation of genes, both in pure bacterial culture and in intracellular bacteria harvested from infected THP-1 macrophages. Therefore, in this study, reverse transcription real-time quantitative PCR (RT-qPCR) was used to investigate the role of MTP on the regulation of genes that modulate lipid metabolism, carbon metabolism and cellular import/transport functions in bacterial culture and intracellular bacteria isolated from infected THP-1 macrophages. The data presented provide further insight into the metabolomic alterations observed in the previous studies [14,15].

## Methods

### Ethics statement and laboratory facilities

The Biomedical Research Ethics Committee of the University of KwaZulu-Natal approved this study (BREC/00002915/2021). The experimental work was conducted in a Biosafety Level 2+ (BSL 2+) laboratory at Medical Microbiology, University of KwaZulu-Natal, Durban South Africa.

### Bacterial strains and growth conditions

*M. tuberculosis* V9124 from the F15/LAM4/KZN family [31] was used as the WT strain in this study. The marked *mtp*-deletion mutant (∆*mtp*) was constructed through specialized transduction of the WT by deleting and replacing the 188 bp region of the *mtp* gene with a γδres-sacB-hyg-γδres cassette [7,32]. This deletion mutant was used to construct its *mtp*-complement strain via electrotransformation with pMV261 [7]. These strains were confirmed to either possess or lack *mtp* prior to experimental assays.

The strains were cultured in Middlebrook 7H9 broth (Difco, Becton–Dickinson, Franklin Lakes, NJ, USA) supplemented with 10% (v/v) oleic albumin dextrose catalase (Becton–Dickinson, Franklin Lakes, NJ, USA), 0.5% (v/v) glycerol (Sigma-Aldrich, St. Louis, MO, USA) and 0.05% Tween-80 (Sigma-Aldrich, St. Louis, MO, USA). Prior to experiments, the ∆*mtp* was cultured in media supplemented with hygromycin B (75 µg ml^−1^), and the *mtp*-complement was cultured with hygromycin B (75 µg ml^−1^) and kanamycin (30 µg ml^−1^). Cultures for c.f.u. were grown on Middlebrook 7H11 plates (Difco, Becton–Dickinson, Franklin Lakes, NJ, USA) similarly supplemented as described above except for the addition of Tween 80. All cultures were incubated at 37 °C for 3–4 weeks (plates) or in a shaking incubator at 100 r.p.m. for 7–8 days (broth).

The three bacterial strains were cultured to late log phase with an OD_600nm_ of ~1 (0.8 to 1 range), equivalent to 1×10^8^ cells per ml^−1^ [33] in 50 ml of supplemented 7H9 (Table S1 Supplementary Material 1). Thereafter, cultured bacteria were pelleted at 4,000*×g* for 15 min at 4 °C (Mikro 200R, Hettich Zentrifugen, Tuttlingen, Germany). The pellets were resuspended in 1 ml TriZol reagent (Ambion, Life Technologies, USA) and transferred into O-ring tubes for storage at −80 °C until RNA was extracted. This experiment was conducted three times, with three replicates per strain.

### Host cell lines, growth conditions and infection with *M. tuberculosis*

Roswell Park Memorial Institute Medium 1640 medium with l-glutamine (Lonza, BioWhittaker, Basel, Switzerland) was supplemented with 10% fetal bovine serum (Biowest, Nuaillé, France) to produce complete cell culture media for the growth of the THP-1 monocytic cell line (ATCC TIB-202). Cells were cultured in T75 cm^2^ tissue culture flasks (Nest Biotechnology, Jiangsu, China) and incubated at 37 °C in 5% CO_2_ in a humidified incubator (Shel Lab CO_2_ Incubator, Cornelius, OR, USA) until 70%–90% confluency.

A total of three biological assays were performed, each with 24 seeded flasks. Forty-eight hours prior to infection, THP-1 monocytes were seeded at a density of 1.25×10^6^ cells per ml^−1^ in a total of 25 ml and were differentiated into macrophages with phorbol 12-myristate 13-acetate (50 ng ml^−1^) (Sigma-Aldrich, St. Louis, MO, USA). The media were changed 24 h later, and macrophages were allowed to rest for 24 h prior to infection.

Bacterial inocula were prepared to an OD_600nm_ of ~1 for each *M. tuberculosis* strain so that a multiplicity of infection (MOI) ratio of ~5:1 bacteria to host was achieved (Table S2 Supplementary Material 1). Macrophage monolayers were rinsed once with phosphate buffered saline (PBS) (Sigma-Aldrich, St. Louis, MO, USA), and complete cell culture media were replaced before infection with the respective bacterial inocula. The flasks were incubated at 37 °C in 5% CO_2_ and 95% humidity for 4 h of infection. Tenfold serial dilutions of bacterial inocula were plated onto Middlebrook 7H11 agar plates to confirm the MOI (Table S2 Supplementary Material 1). Following the 4 h infection, the monolayers were immediately washed with PBS and lysed with 0.25% Triton X-100 (Millipore, Merck, Darmstadt, Germany) for 20 min. Lysates were pelleted and rinsed with PBS before combining the six replicates of each *M. tuberculosis* strain into two samples for storage in TriZol reagent as described above (three flasks into one sample). Of the 24 flasks, six were used for plating out tenfold serial dilutions for the determination of c.f.u. to ascertain the reproducibility of infection as well as calculate the invasion of *M. tuberculosis* (Tables S3, S4 and S5 Supplementary Material 1).

### RNA extraction, DNase treatment and cDNA conversion

RNA extractions were performed according to [33], with the use of a Precellys 24 lysis and homogenization machine (Bertin Technologies, Montigny-le-Bretonneux, France) and the addition of an extra 70% ethanol wash. RNA concentrations and purities were evaluated via optical density readings and denaturing gels for bacterial culture samples (Table S6 and Fig. S1 Supplementary Material 1) and intracellular bacterial samples (Table S7 and Fig. S2 Supplementary Material 1). A single sample for each strain from each biological assay was selected for DNase treatment and cDNA synthesis. The RNA was standardized to 100 µg µl^−1^ prior to DNase treatment (Thermo Fisher Scientific, Waltham, MA, USA) and converted to cDNA with the high-capacity cDNA reverse transcription kit (Roche Applied Sciences, Penzberg, Germany) as per the manufacturer’s instructions. The cDNA quality, concentration and purity for bacterial culture samples (Table S8 and Fig. S3 Supplementary Material 1) and intracellular bacterial samples (Table S9 and Fig. S4 Supplementary Material 1) were considered prior to RT-qPCR.

### RT-qPCR

Primer efficiencies were tested, and PCR conditions were optimized for all primer sets prior to experiments. The SsoAdvanced Universal SYBR Green Supermix kit (Bio-Rad, Hercules, CA, USA) was used to perform RT-qPCR using a total reaction volume of 10 µl, in a 7500 RT-qPCR Detection System (Applied Biosystems, Foster City, CA, USA). Cycling conditions were as follows for a total of 40 cycles: holding stage at 95 °C for 30 s and cycling stage at 95 °C for 5 s with specific annealing temperatures for 30 s (Table S10 Supplementary Material 1). Melt curve analysis was measured as continuous fluorescence set at 95 °C for 15 s, 60 °C for 1 min, 95 °C for 30 s and 60 °C for 15 s. The resulting gene expression data was normalized using 16S rRNA and was analysed by absolute quantification using the standard curve method. Three biological replicates and four technical replicates were performed for each strain and gene.

### Data analysis

**Fig. 1.**
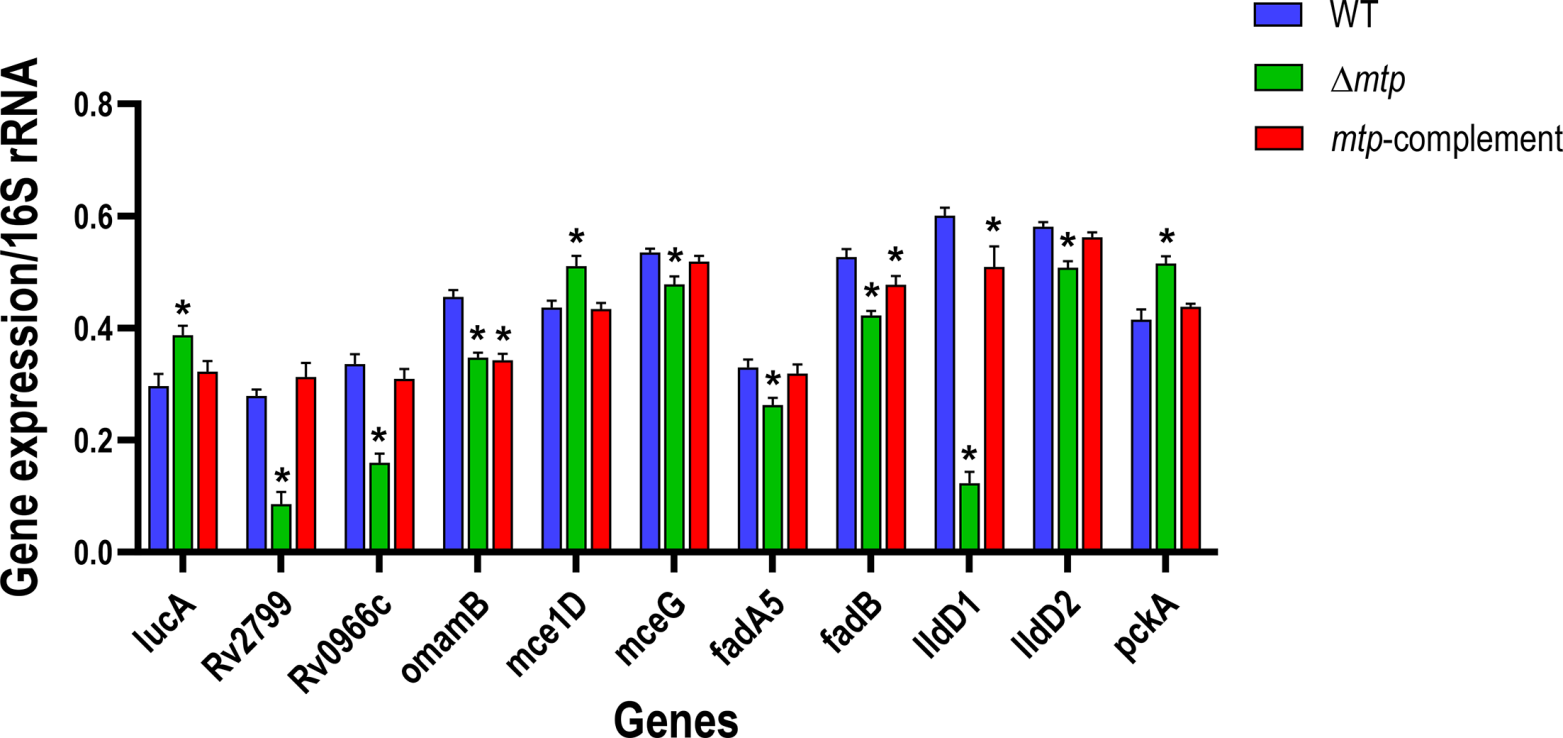
Bacterial gene expression levels of the three mycobacterial strains grown in Middlebrook 7H9 broth culture. Absolute quantification of gene expression was determined through RT-qPCR of the bacterial strains (WT V9124, Δ*mtp* and *mtp*-complement). Three biological assays and four technical repeats were conducted for each of the 11 selected genes (*lucA*, *Rv2799*, *Rv0966c*, *omamB*, *mce1D*, *mceG*, *fadA5*, *fadB*, *lldD1*, *lldD2* and *pckA*). Gene expression levels were normalized to the housekeeping 16S rRNA for each gene of interest and were represented by standard error mean (SEM) bars. Data were analysed by one-way ANOVA and Tukey’s test with statistical significance considered when the adjusted *P*≤0.05 (indicated by * to denote significance relative to WT). Significant differences between WT and Δ*mtp* were seen for *lucA* (*P*=0.005), *Rv2799* (*P*<0.001), *Rv0966c* (*P*<0.001), *omamB* (*P*<0.001), *mce1D* (*P*=0.003), *mceG* (*P*=0.002), *fadA5* (*P*=0.007), *fadB* (*P*<0.001), *lldD1* (*P*<0.001), *lldD2* (*P*<0.001) and *pckA* (*P*<0.001). Significant differences between WT and *mtp-*complement were seen for *omamB* (*P*<0.001), *fadB* (*P*<0.034) and *lldD1* (*P*<0.040).

**Fig. 2.**
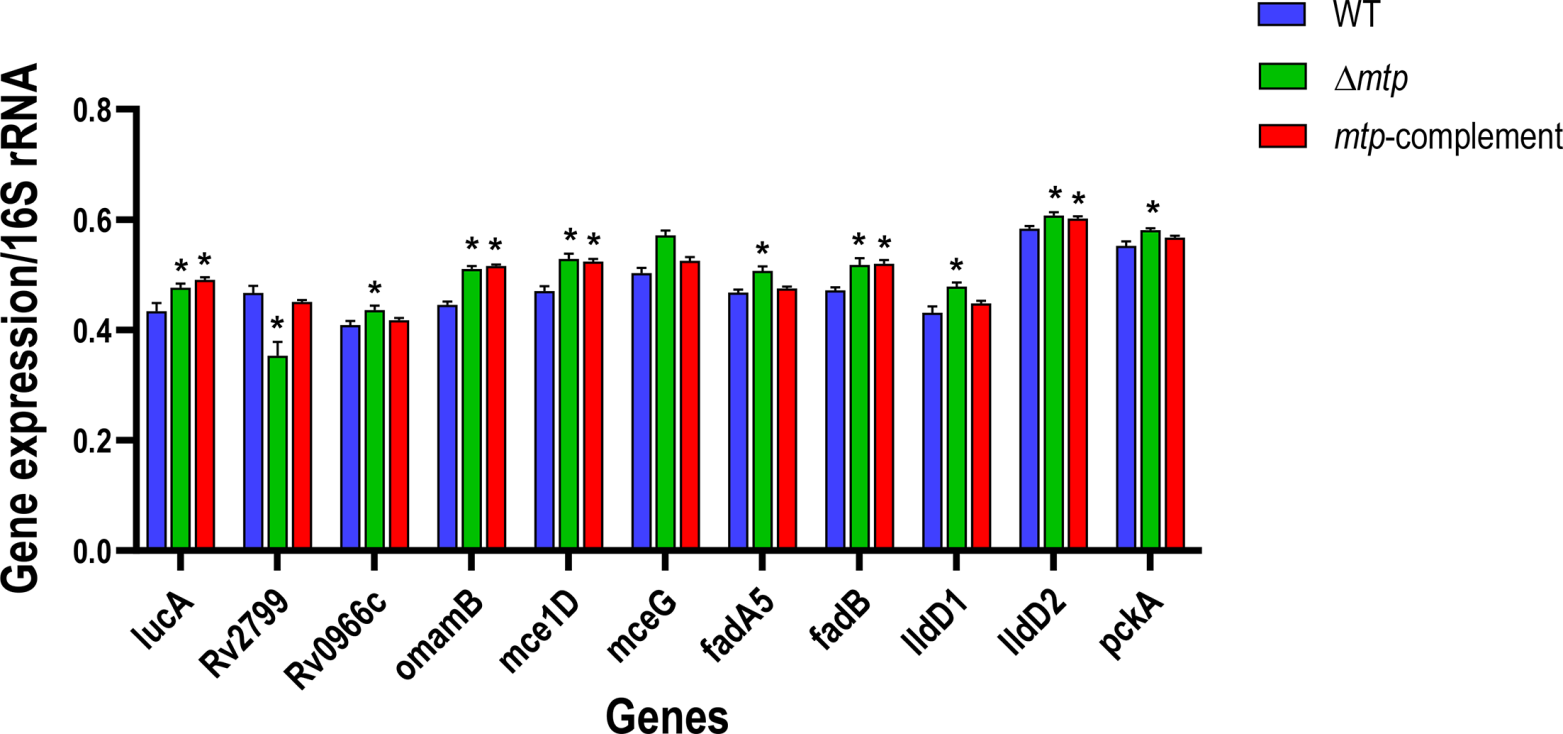
Intracellular gene expression levels of the three mycobacterial strains during early infection of THP-1 macrophages. Absolute quantification of gene expression was determined through RT-qPCR of the intracellular bacterial strains (WT V9124, Δ*mtp* and *mtp*-complement) isolated from the THP-1 macrophage infection model. Three biological assays and four technical repeats were conducted for each of the 11 selected genes (*lucA*, *Rv2799*, *Rv0966c*, *omamB*, *mce1D*, *mceG*, *fadA5*, *fadB*, *lldD1*, *lldD2* and *pckA*). Gene expression levels were normalized to the housekeeping 16S rRNA for each gene of interest and were represented by standard error mean (SEM) bars. Data were analysed by one-way ANOVA and Tukey’s test with statistical significance considered when the adjusted *P*≤0.05 (indicated by * to denote significance relative to WT). Significant differences between WT and Δ*mtp* were seen for *lucA* (*P*=0.011), *Rv2799* (*P*<0.001), *Rv0966c* (*P*=0.020), *omamB* (*P*<0.001), *mce1D* (*P*<0.001), *fadA5* (*P*<0.001), *fadB* (*P*=0.002), *lldD1* (*P*=0.001), *lldD2* (*P*=0.005) and *pckA* (*P*=0.002). Significant differences between WT and *mtp-*complement were seen for *lucA* (*P*=0.001), *omamB* (*P*<0.001), *mce1D* (*P*<0.001), *fadB* (*P*=0.001) and *lldD2* (*P*=0.030).

**Fig. 3.**
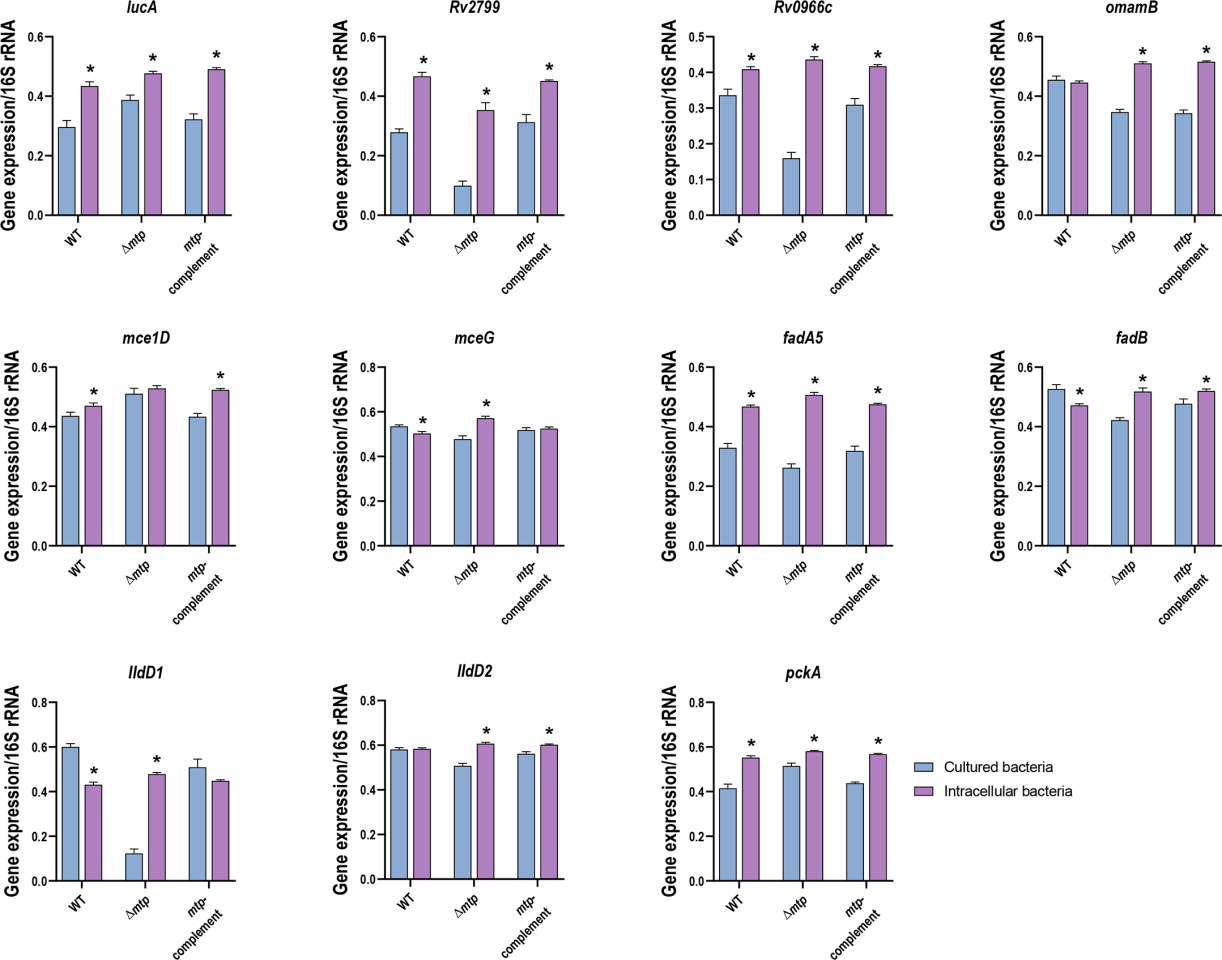
Gene expression comparison between cultured *M. tuberculosis* and intracellular *M. tuberculosis* from THP-1 macrophage infections. Absolute quantification of gene expression was determined through RT-qPCR of the cultured bacterial strains and bacterial RNA isolated from intracellular bacteria (WT V9124, Δ*mtp* and *mtp*-complement strains). Three biological assays and four technical repeats were conducted for each of the 11 selected genes (*lucA*, *Rv2799*, *Rv0966c*, *omamB*, *mce1D*, *mceG*, *fadA5*, *fadB*, *lldD1*, *lldD2* and *pckA*). Gene expression levels were normalized to the housekeeping 16S rRNA for each gene of interest and are represented by standard error mean (SEM) bars. Data were analysed by parametric unpaired t-test with statistical significance considered when the *P*≤0.05 (indicated by * to denote significance between models of the same strain). Significant differences between cultured WT and intracellular WT were seen for *lucA* (*P*<0.001), *Rv2799* (*P*<0.001), *Rv0966c* (*P*=0.001), *mce1D* (*P*=0.040), *mceG* (*P*=0.011), *fadA5* (*P*<0.001), *fadB* (*P*=0.002), *lldD1* (*P*<0.001) and *pckA* (*P*<0.001). Significant differences between Δ*mtp* and intracellular Δ*mtp* were seen for *lucA* (*P*<0.001), *Rv2799* (*P*<0.001), *Rv0966c* (*P*<0.001), *omamB* (*P*<0.001), *mceG* (*P*<0.001), *fadA5* (*P*<0.001), *fadB* (*P*<0.001), *lldD1* (*P*<0.001), *lldD2* (*P*<0.001) and *pckA* (*P*<0.001). Significant differences between *mtp-*complement and intracellular *mtp-*complement were seen for *lucA* (*P*<0.001), *Rv2799* (*P*<0.001), *Rv0966c* (*P*<0.001), *omamB* (*P*<0.001), *mce1D* (*P*<0.001), *fadA5* (*P*<0.001), *fadB* (*P*=0.021), *lldD2* (*P*=0.001) and *pckA* (*P*<0.001).

The standard curve parameters for each gene were used to calculate absolute transcript numbers for each run with the equation: *y=mx+* c, in which *y* was the *C*_*t*_ value, *m* was the slope, *c* was the *y*-intercept and *x* was the transcript number. The housekeeping gene (16S rRNA) was used to normalize the individual transcript numbers (ratio=gene transcript number/16S rRNA transcript number). The resulting data were tested for normality by a Shapiro–Wilk test in which parametric data will have a *P-*value≥0.05. For multiple comparisons, the normalized data were used to conduct one-way ANOVA with Tukey’s test for correcting for multiple comparisons (post hoc analysis) for each gene. This was done to compare the effect of the absence/presence of MTP on gene expression among *M. tuberculosis* strains from bacterial cultures (Figs 1 and S5a Supplementary Material 1) and intracellular *M. tuberculosis* from infected THP-1 macrophages (Figs 2 and S5b Supplementary Material 1) in which the *P*-values≤0.05 were considered significant. Additionally, cultured bacterial and intracellular bacterial gene expression was compared for each gene by parametric unpaired t-test with statistical significance considered when the *P*≤0.05 (Fig. 3). All the statistical analyses were performed on GraphPad Prism version 8.0.

## Results

### MTP upregulates the genes associated with the uptake and breakdown of lipids and lactate during the late log growth phase of *M. tuberculosis* in liquid culture

The absolute transcript numbers of cultured bacteria were determined through RT-qPCR, and the expression of all 11 genes were statistically significantly different between the WT and Δ*mtp* strains (Fig. 1). The gene expressions of *lucA* (*P*=0.005), *mce1D* (*P*=0.003) and *pckA* (*P*<0.001) were significantly higher in Δ*mtp* than the WT and *mtp*-complement, but similar between the latter two strains. This indicates that under optimum growth conditions, during the late log phase of *M. tuberculosis* growth, MTP downregulates *lucA*, *mce1D* and *pckA*. This is expected to affect the putative components of fatty acid transporters and the rate of gluconeogenesis by resulting in a reduction of flow through both these processes in the WT and *mtp*-complement.

However, the gene expressions of *Rv2799* (*P*<0.001), *Rv0966c* (*P*<0.001), *mceG* (*P*=0.002)*, fadA5* (*P*=0.007) and *lldD2* (*P*<0.001) were significantly lower in Δ*mtp* than in the WT and *mtp*-complement, but similar in the latter two strains. Therefore, in the presence of MTP, these putative components of the fatty acid transporters are active along with fatty acid oxidation during exponential growth of *M. tuberculosis*. Hence, *M. tuberculosis* readily uses lipids and lactate during growth.

Although *omamB* expression was significantly lower in Δ*mtp* than the WT (*P*<0.001), there was no significant difference between Δ*mtp* and *mtp*-complement. Moreover, the *mtp*-complement was significantly different from the WT for *omamB* (*P*<0.001). The expressions of *fadB* and *lldD1* were significantly different for all three strains, with the lowest seen in the Δ*mtp* and the highest in the WT. The *mtp*-complement displayed gene expression levels between that of the Δ*mtp* and WT for *fadB* and *lldD1*. The majority of the gene expression for WT and *mtp-*complement were similar except for o*mamB*. Overall, the *mtp-*complement displayed gene expression levels between the expression profiles of the WT and Δ*mtp*. From these gene expression trends, it is deduced that the complementation of *mtp* by a non-integrating plasmid resulted in partial restoration of MTP. Furthermore, MTP played a role in upregulating 8 of the 11 genes investigated in the cultured bacterial model, as is visualized by the WT displaying higher gene expression for *Rv2799*, *Rv0966c*, *omamB*, *mceG*, *fadA5*, *fadB, lldD1* and *lldD2* relative to Δ*mtp*.

### MTP downregulates the genes involved in the uptake and breakdown of lipids and lactate of intracellular *M. tuberculosis* during early infection

Ten of the 11 genes investigated displayed statistically significant differences between the WT and Δ*mtp* strains (Fig. 2). The expression of four genes, *Rv0966c* (*P*=0.020), *fadA5* (*P*<0.001), *lldD1* (*P*=0.001) and *pckA* (*P*=0.002), were significantly higher in Δ*mtp* than in the WT and *mtp*-complement strains, but similar for the latter two strains. The gene expression of *Rv2799* (*P*<0.001) was significantly lower in Δ*mtp* than in the WT and *mtp*-complement, while no significant difference was observed between the latter two strains. The gene expressions of *lucA*, *omamB*, *mce1D*, *fadB* and *lldD2* were significantly higher in Δ*mtp* and *mtp-*complement than in the WT. For *lucA* and *omamB,* the lowest gene expression was seen in the WT, followed by Δ*mtp* and then *mtp-*complement with the highest gene expressions. For *mce1D, fadB* and *lldD2,* the lowest gene expression was seen in the WT, followed by *mtp-*complement and then Δ*mtp*. The gene expression of *mceG* was not significantly different amongst all three strains*;* however, the highest gene expression was observed in the Δ*mtp,* followed by *mtp-*complement and then WT.

The data once again revealed partial restoration of MTP via complementation. Relative to the WT, the intracellular Δ*mtp* displayed a higher expression for all the investigated genes, except *Rv2799*. Holistically, these data indicate that MTP plays a role in the downregulation of these genes in intracellular *M. tuberculosis* during the initial infection process.

### MTP regulates the expression of genes involved in fatty acid import and lactate oxidation of intracellular *M. tuberculosis*

When viewed separately, the expression levels of 7 of 11 genes (*Rv0966c*, *omamB*, *mceG*, *fadA5*, *fadB*, *lldD1* and *lldD2*) showed an inverse trend between the two models in the Δ*mtp* groups. However, to evaluate changes in gene expression during the initial stages of infection, cultured bacterial gene expression was compared to the intracellular gene expression for each strain (Fig. 3). The data revealed that the intracellular bacteria displayed a significantly higher expression of *lucA* (*P*<0.001), *Rv2799* (*P*<0.001), *Rv0966c* (*P*=0.001), *fadA5* (*P*<0.001) and *pckA* (*P*<0.001) in all three strains relative to the cultured bacterial counterparts. These results indicate that the genes encoding these putative components of lipid transporters, which facilitate the import of both fatty acids and cholesterol, as well as β-oxidation and gluconeogenic carbon flow, are not dependent on the MTP adhesin. Rather, the upregulation of these genes is likely triggered by the entry of *M. tuberculosis* into the THP-1 macrophage environment to allow the pathogen to acquire the necessary nutrients. Although there is no statistical evidence, the change in expression levels of these genes in Δ*mtp* appears to have an altered change in expression levels between the two models (they either exhibit the largest change or smallest change), relative to the MTP-proficient strains.

Significant differences in the expression of *omamB*, *mce1D*, *mceG*, *fadB*, *lldD1* and *lldD2* were observed between cultured and intracellular bacteria. Significantly, lower expression of *fadB* was observed in the intracellular WT (*P*=0.002) compared to the broth-grown WT. In contrast, significantly higher *fadB* was expressed by the intracellular Δ*mtp* (*P*<0.001) and intracellular *mtp*-complement (*P*=0.021) strains compared to their respective cultured bacterial groups. Genes such as *omamB* (*P*<0.001) and *lldD2* (*P*<0.001) were expressed at significantly higher levels in the intracellular Δ*mtp* and *mtp-*complement strains, while no significant difference was exhibited by the WT in both models. The WT (*P*=0.040) and *mtp-*complement (*P*<0.001) demonstrated significant differences in *mce1D* expression*,* with higher gene expression in the intracellular group, whereas the trend seen in Δ*mtp* differed from the WT and *mtp-*complement strains as it showed no difference in *mce1D* expression between the bacterial culture and intracellular bacteria. The intracellular WT expressed lower levels of *mceG* and *lldD1* (*P*=0.011 and *P*<0.001, respectively). In contrast, significantly higher expression of both these genes was displayed by the intracellular Δ*mtp* (*P*<0.001 for both), with the *mtp-*complement showing no statistical difference between the bacterial groups.

Thus, the findings demonstrate that in the presence of MTP, *mceG* and *lldD1* are downregulated, while *mce1D* is upregulated in intracellular *M. tuberculosis*. This indicates that MTP regulates the expression of the lactate dehydrogenase gene, *lldD1*, and fatty acid import genes, *mce1D* and *mceG,* of *M. tuberculosis* during the first 4 h of infection.

## Discussion

The unique and conserved MTP adhesin has been identified as a potential target for TB treatment strategies [5]. Numerous *M. tuberculosis* virulence studies have revealed that MTP not only acts as an adhesin and invasin to host cells [8,34] but also modulates host immune responses and metabolism during infection [9,12, 14,17]. Given that *mtp* is situated amongst intermediary metabolism genes [4], clear differences in metabolite concentrations of pure bacterial culture and infection models were proposed to be attributed to the presence or absence of MTP [14,15, 17]. Untargeted metabolomics had previously demonstrated that Δ*mtp* displayed an accumulation of intermediates of fatty acid metabolism in both late log phase bacterial culture and THP-1 macrophage infection models [14,15, 17]. Additionally, perturbations were observed for metabolites such as lactate and intermediates of central carbon metabolism [14].

In the current study, expression levels of *M. tuberculosis* genes involved in lipid transport, gluconeogenic carbon flow, β-oxidation and lactate oxidation were evaluated by absolute quantification using the standard curve method for RT-qPCR. *M. tuberculosis* was isolated from broth culture during the late log phase of growth and from cell lysates of THP-1 macrophages following 4 h of infection. The results highlighted the differential gene expression, firstly, among the three different cultures of *M. tuberculosis* strains (WT, Δ*mtp* and *mtp*-complement) (Fig. 1), secondly, among intracellular bacteria of the three strains (Fig. 2) and, thirdly, between bacteria grown in culture medium and intracellular bacteria (Fig. 3). Most of the genes investigated were downregulated in the exponentially growing MTP-deficient *M. tuberculosis* relative to the MTP-proficient strains. Contrastingly, most of these genes were upregulated in the MTP-deficient intracellular *M. tuberculosis*. At a glance, MTP appears to be more influential on gene expression levels of *M. tuberculosis* grown in broth culture than that extracted from the intracellular space (Fig. S5 Supplementary Material 1). Therefore, a comparison of the gene expression levels between these two models was statistically evaluated and revealed that MTP is significantly associated with the regulation of the intracellular pathogen’s genes involved in lipid import (*mce1D* and *mceG*) and lactate oxidation (*lldD1*).

*M. tuberculosis* is a proficient pathogen as it quickly adapts to changes in its microenvironment and is capable of surviving on a plethora of nutrients. Although *M. tuberculosis* can survive extracellularly, much of its life cycle is committed to growing intracellularly, where nutrient availability is known to fluctuate [35,36]. Moreover, *M. tuberculosis* is adept at co-catabolizing different carbon sources [37]. The acquisition and assimilation of multiple carbon sources such as sugars, amino acids, host‐derived fatty acids and cholesterol provide the pathogen with sources of nitrogen, carbon and energy during host infection [38].

### Lipid import via Mce1 transport protein and β-oxidation of fatty acids modulated by MTP alters the pathogenicity of intracellular *M. tuberculosis*

Cholesterol is known to accumulate at the site of infection and is essential for mycobacterial entry into macrophages [39]. Accumulation of cholesterol within macrophages occurs as a result of *M. tuberculosis* triggering the host to increase the uptake and enhance *de novo* cholesterol synthesis, inhibit the degradation of cholesterol esters and impede cholesterol efflux [40]. The accumulation of cholesterol promotes the formation of lipid droplets and foam cells, ultimately facilitating the persistent survival of the pathogen [40]. Moreover, when varying nutrient availability is encountered during infection, *M. tuberculosis* heavily relies on its adaptability to utilize host lipids for the growth and structural integrity of the mycobacterial cell wall by the formation of complex lipids [22]. This subsequently provides protection against host immune responses and antibiotics.

Each multi-protein Mce transporter is known to import different lipids, with Mce1 chiefly transporting fatty acids and Mce4 dedicated to importing cholesterol [22]. The shared protein, LucA, interacts with both Mce1 and Mce4 subunits, thus facilitating the coordinated import of lipids [22]. Mycobacterial Mce transporters comprise cytoplasmic ATPase (MceG) and multiple Mce-associated membrane (Mam) and orphaned Mam (Omam) proteins [41]. These complex import proteins are integral to *M. tuberculosis* pathogenicity, and their regulation determines the outcome of infection. Therefore, in this study, *lucA* and genes encoding putative components of the Mce1 transporter (*Rv2799*, *Rv0966c*, *mce1D*, *mceG* and *omamB*) were investigated in the absence/presence of MTP.

Cultured and intracellular Δ*mtp* had higher expression of *lucA* relative to the WT (Figs1  2). When bacterial culture and intracellular Δ*mtp* were compared, *lucA* exhibited the smallest difference in expression levels between the two models (Fig. 3). Studies have demonstrated that mutations in *lucA* lead to impaired growth of *M. tuberculosis* in environments where cholesterol is the primary carbon source [22]. Hence, MTP regulates the expression of *lucA* in response to the change in environment, allowing the pathogen to adequately adapt to the available nutrients.

Of the five genes associated with putative components of the Mce1 fatty acid transporter, four (*Rv2799*, *Rv0966c*, *omamB* and *mceG*) were expressed at lower levels in the bacterial culture of Δ*mtp* relative to the WT (Fig. 1). These findings suggest that the lack of MTP results in the potential reduction of fatty acid import in bacterial culture. Moreover, lower expression levels were also observed in both β-oxidation genes investigated (*fadA5* and *fadB*) in the bacterial culture of Δ*mtp* (Fig. 1), suggesting that MTP plays a role in fatty acid oxidation. Hence, MTP facilitates the upregulation of genes involved in fatty acid importer Mce1 and the FadA–FadB complex involved in *β*-oxidation within *M. tuberculosis*.

Moreover, the reduced expression of fatty acid import and oxidation genes, coupled with the previously reported fatty acid accumulation in Δ*mtp* culture [15], is proposed to additionally be influenced by *de novo* fatty acid synthesis. Bacterial *de novo* fatty acid synthesis is achieved by type I fatty acid synthase (FAS-I) and type II fatty acid synthase (FAS-II). The multifunctional enzyme, FAS-I, initiates *de novo* synthesis of medium-chain fatty acids from acetyl-CoA, while FAS-II is responsible for their elongation into longer-chain fatty acids, such as mycolic acids, which critically contribute to the structural integrity and impermeability of the mycobacterial cell envelope [42,44]. Regulation of *M. tuberculosis* fatty acid synthases is complex and involves various transcriptional regulators such as the transcriptional repressor Mce3R [45] and the essential activator FasR [46]. Therefore, further investigation of *de novo* fatty acid regulation and synthesis is required to confirm if *M. tuberculosis* potentially overcompensates for the lack of import and oxidation of extracellular lipids in the absence of MTP. Currently, data suggest that MTP aids *M. tuberculosis* in importing and breaking down fatty acids to meet growth requirements.

Interestingly, the higher expression levels of *Rv0966c*, *omamB, mce1D*, *fadA5* and *fadB* (Fig. 2) of intracellular Δ*mtp* indicate that the previously detected accumulation of lipids within the Δ*mtp* infected THP-1 macrophages mainly encompassed the host-derived fatty acids [14]. This deduction is supported by studies that demonstrated that under healthy conditions, *M. tuberculosis* oxygen consumption was one-tenth of the macrophage respiration [47]. Therefore, the contribution of *M. tuberculosis*-derived metabolites to the differential concentrations detected was negligible in comparison to their influence on host activities and the microenvironment [14].

The understanding that *M. tuberculosis* reprogrammes host metabolism to guarantee a continuous supply of essential lipids for its long-term persistence *in vivo* has recently been questioned [48]. Evidence from immunological and microbiological studies has given rise to the new concept that macrophages metabolically adapt to *M. tuberculosis* infection by accumulating fatty acids in the cytoplasm. This potentiates antimycobacterial host responses and influences *M. tuberculosis* to transition into a fat-saving survival mode [48]. Therefore, the previously observed higher fatty acid levels in the Δ*mtp* infected THP-1 macrophage [14], together with the higher expression of intracellular *M. tuberculosis* lipid import and oxidation genes (Fig. 2), indicate that when MTP is absent, the macrophage gravitates towards an antimicrobial response.

### The rate of lactate oxidation and gluconeogenic carbon flow are regulated by MTP

The evolutionary significance of the putative lactate dehydrogenase encoded by *lldD1* is underscored by its conservation in mycobacterial species and its sequence homology to other lactate dehydrogenases [49]. Additionally, *lldD1* aids in the synthesis of the redox cofactor, mycofactocin, which is important for metabolism [50]. Mycofactocin is essential for *M. tuberculosis* growth in the presence of lactate [50]. Thus, *lldD1* is not merely a redundant gene but is a vital component of the metabolic machinery adapted by the pathogen to facilitate metabolic flexibility within the hostile environment of the host [50].

Although demonstrated to be responsible for lactate oxidation [29], the multifunctional *lldD2* is indispensable to *M. tuberculosis*. This gene confers resistance to oxidative stress in addition to metabolic elasticity and virulence within the intracellular niche [51]. Oxidative stress is imposed on *M. tuberculosis* by the host in its efforts to clear out infection. In doing so, *lldD2* manages and maintains the redox balance, which is vital for *M. tuberculosis* survival against reactive oxygen species generated by macrophages in a pro-inflammatory response [51]. The expression of genes involved in cell wall lipid metabolism and virulence systems, such as ESX-1, is also influenced by *lldD2* [51].

The lactate dehydrogenases (*lldD1* and *lldD2*) in the Δ*mtp* displayed the lowest gene expression levels for bacterial culture (Fig. 1) and showed the highest gene expression levels in the intracellular bacteria (Fig. 2). This demonstrates that the mutant did not utilize much of the available lactate in culture but attempted to use the excessively available lactate during infection. Macrophages generate lactate to modulate immune responses [52]. Hence, the disproportionately available lactate previously detected in Δ*mtp* infected THP-1 macrophages [14] is an indication of the macrophage’s response to infection with Δ*mtp* and not an indication of the inability of *M. tuberculosis* to use lactate as a carbon source in the absence of MTP. Therefore, MTP potentially controls lactate oxidation of *M. tuberculosis* to maintain expression levels of *lldD2* or downregulate expression of *lldD1,* particularly when within the host to avoid effector cell detection.

Given that *pckA* is crucial for gluconeogenesis and anaplerotic pathways, studies have shown that upregulation of this gene was found in the presence of fatty acids such as palmitate [53]. This reflects the pivotal function of *pckA* in the pathogen’s adaptability to fluctuating nutrient availability during infection. Furthermore, *pckA* was described as a virulence factor since the absence of PEPCK led to impaired gluconeogenesis and subsequently affected energy production and the survival of bacteria [53]. This gene is also involved in resistance to oxidative stress and facilitates the synthesis of key metabolites that are necessary for mycolic acid and lipid synthesis, which contribute to bacterial structural integrity [54,55].

In the current study, the highest levels of *pckA* expression were observed in the Δ*mtp* for both bacterial culture (Fig. 1) and intracellular bacteria (Fig. 2). Furthermore, an increase in intracellular bacterial gene expression of *pckA* was observed in all bacterial strains, with Δ*mtp* displaying the smallest change in expression (Fig. 3), indicative of the bacterium’s fitness. These findings demonstrate that gluconeogenic carbon flow is not dependent on the presence of MTP. Rather, the rate of *M. tuberculosis* gluconeogenic carbon flow is influenced by MTP during exponential growth and replication.

### MTP shapes the extent of cultured and intracellular bacterial gene regulation

The analysis of the pure bacterial culture and intracellular bacterial gene expression levels revealed that the change in environment resulted in an overall trend of an atypical response of Δ*mtp* compared to that of the WT and *mtp-*complement (Fig. 3). The gene expression levels were either drastically increased or minimally changed in the mutant strain. This indicates that MTP may additionally be associated with maintaining the bacterial metabolic regulation in order to ensure successful infection of THP-1 macrophages and, thus, validates previous findings [8,14, 34, 56] of MTP as a virulence factor of *M. tuberculosis*. While data from this study are restricted to a 4 h timepoint, previous studies have demonstrated the virulence attributes of MTP from the time of infection to 21 days post-infection [10,12, 56].

This study was limited to three bacterial strains, and the complemented strain used in this study only displayed partial transcriptomic restoration, given the nature of its construct. A select few genes were investigated; hence, the data presented here are merely a preview of the transcriptomic alterations caused by the MTP or the lack thereof. The changes seen in gene expression levels for the two models used in this study may not be representative of a larger and more complex pathogen–host system and interactions. Nevertheless, the information presented here provides new knowledge on the adaptability of *M. tuberculosis* to exploit different strategies to survive and succeed as an intracellular pathogen.

## Conclusion

Removal of the MTP from *M. tuberculosis* culminates in transcriptional alterations observed in bacterial culture, as well as intracellular bacteria isolated from infected THP-1 macrophages. Adjustments to lipid import, β-oxidation and lactate oxidation of *M. tuberculosis* indicated that MTP may be associated with the modulation of multiple functions during early infection. Therefore, MTP potentially acts as a modulator of genes that contribute to the metabolism and metabolic flexibility of *M. tuberculosis*. This study provided evidence that corroborated previous metabolomic outcomes and highlighted the bacterial molecular mechanisms influenced by MTP. In conclusion, MTP significantly contributes to the success of *M. tuberculosis* as an intracellular pathogen. By shifting the focus of TB intervention strategies towards intercepting the MTP interaction with the host, it may be possible to offset *M. tuberculosis* pathogenicity and confer an advantage to the host.

## supplementary material

10.1099/jmm.0.001994 Supplementary Material 1.

## Abbreviations

FAS-I: type I fatty acid synthase
FAS-II: type II fatty acid synthase
Mam: Mce-assosiated membrane
Mce: mammalian cell entry
MDR-TB: multidrug-resistant tuberculosis
MOI: multiplicity of infection
MTP: *Mycobacterium tuberculosis* curli pili
Omam: orphaned Mce-assosiated membrane
ORF: open reading frame
PBS: phosphate buffered saline
PEPCK: phosphoenolpyruvate carboxykinase
RR-TB: rifampicin resistant tuberculosis
RT-qPCR: reverse transcription real-time quantitative PCR
TB: tuberculosis
WT: wildtype
