# Supplementary material for: Differential expression of genes associated with lipid import, β-oxidation and lactate oxidation induced by Mycobacterium tuberculosis curli pili in broth culture compared to intracellular bacilli within THP-1 macrophages
Source: J Med Microbiol. 2025 Mar 31;74(3):001994. doi: 10.1099/jmm.0.001994 (PMC11956070; doi:10.1099/jmm.0.001994)
Supplement: Supplementary Material 1. [file jmm-74-01994-s001.pdf]

Table S1. Average optical density readings at 600_{nm} of cultured *M. tuberculosis* strains for the three biological assays.

**Strain**	**Biological assay 1 (OD_{600nm})**	**Biological assay 2 (OD_{600nm})**	**Biological assay 3 (OD_{600nm})**
WT	0.824	0.820	0.813
Δ*mtp*	0.821	0.802	0.898
mtp-complement	0.855	0.840	0.876

WT, wildtype *M. tuberculosis* V9124; Δ*mtp, M. tuberculosis mtp*-deletion mutant; *mtp*-complement, *M. tuberculosis mtp*-complement.

Table S2. Multiplicity of infection (MOI) of the three biological assays for the THP-1 macrophage infections.

**Strain**	**OD_{600nm}**	**Dilution**	**Replicate 1**	**Replicate 2**	**Replicate 3**	**Average**	**CFU/mL**	**MOI**
Biological assay 1 WT	1.009	10^{-2}	Lawn	Lawn	Lawn	-	-	-
		10^{-3}	>200	>200	>200	-	-	-
		10^{-4}	104	122	107	111.00	1.1×10^8	5.28
		10^{-5}	23	19	17	-	-	-
Biological assay 1 Δ*mtp*	1.036	10^{-2}	Lawn	Lawn	Lawn	-	-	-
		10^{-3}	>200	>200	>200	-	-	-
		10^{-4}	124	122	113	119.67	1.2×10^8	5.744
		10^{-5}	42	22	31	-	-	-
Biological assay 1 *mtp*-complement	1.012	10^{-2}	Lawn	Lawn	Lawn	-	-	-
		10^{-3}	>200	>200	>200	-	-	-
		10^{-4}	109	98	89	98.67	9.9×10^7	4.74
		10^{-5}	12	28	9	-	-	-
Biological assay 2 WT	1.016	10^{-2}	Lawn	Lawn	Lawn	-	-	-
		10^{-3}	>200	>200	>200	-	-	-
		10^{-4}	101	97	106	101.33	1.0×10^8	4.86
		10^{-5}	11	9	7	-	-	-
Biological assay 2 Δ*mtp*	1.006	10^{-2}	Lawn	Lawn	Lawn	-	-	-
		10^{-3}	>200	>200	>200	-	-	-
		10^{-4}	121	113	90	108.00	1.1×10^8	5.18
		10^{-5}	8	12	15	-	-	-
Biological assay 2 *mtp*-complement	1.009	10^{-2}	Lawn	Lawn	Lawn	-	-	-
		10^{-3}	>200	>200	>200	-	-	-
		10^{-4}	107	99	85	97.00	9.7×10^7	4.66
		10^{-5}	10	17	9	-	-	-
Biological assay 3 WT	0.940	10^{-2}	Lawn	Lawn	Lawn	-	-	-
		10^{-3}	>200	>200	>200	-	-	-
		10^{-4}	109	98	82	96.33	9.6×10^7	4.62
		10^{-5}	5	8	14	-	-	-
Biological assay 3 Δ*mtp*	1.002	10^{-2}	Lawn	Lawn	Lawn	-	-	-
		10^{-3}	>200	>200	>200	-	-	-
		10^{-4}	117	120	111	116.00	1.2×10^8	5.57
		10^{-5}	20	15	14	-	-	-
Biological assay 3 *mtp*-complement	1.017	10^{-2}	Lawn	Lawn	Lawn	-	-	-
		10^{-3}	>200	>200	>200	-	-	-
		10^{-4}	124	98	86	102.67	1.0×10^8	4.93
		10^{-5}	12	8	9	-	-	-

WT, wildtype *M. tuberculosis* V9124; Δ*mtp, M. tuberculosis mtp*-deletion mutant; *mtp*-complement; MOI, multiplicity of infection; CFU, colony forming unit.

$$CFU/mL = \frac{(\text{average number of colonies} \times \text{dilution factor})}{\text{volume plated out in mL (0.01)}}$$

$$MOI = \frac{\text{Total number of bacterial cells}}{\text{Total number of THP−1 cells}} = \frac{(\text{CFU/mL} \times \text{total volume of inoculum in mL (1.5 mL)})}{(\text{Number of cells } (1.25\times10^6)\times\text{volume (25 mL)})}$$

Table S3. Colony forming units (CFUs) of the biological assay 1 of the THP-1 macrophage infections.

Strain	Dilution	Replicate 1	Replicate 2	Replicate 3	Average	CFU/mL	Average CFU/mL
Biological assay 1 WT 1	Neat	Lawn	Lawn	Lawn	-	-	1.2×10^6
	10^{-1}	>200	>200	>200	-	-	
	10^{-2}	120	117	125	120.67	1.2×10^6	
	10^{-3}	11	10	12	-	-	
Biological assay 1 WT 2	Neat	Lawn	Lawn	Lawn	-	-	
	10^{-1}	>200	>200	>200	-	-	
	10^{-2}	128	114	115	119.00	1.2×10^6	
	10^{-3}	17	12	18	-	-	
Biological assay 1 Δ*mtp* 1	Neat	Lawn	Lawn	Lawn	-	-	1.1×10^6
	10^{-1}	>200	>200	>200	-	-	
	10^{-2}	98	107	102	102.33	1.0×10^6	
	10^{-3}	3	6	8	-	-	
Biological assay 1 Δ*mtp* 2	Neat	Lawn	Lawn	Lawn	-	-	
	10^{-1}	>200	>200	>200	-	-	
	10^{-2}	99	114	119	110.67	1.1×10^6	
	10^{-3}	9	16	13	-	-	
Biological assay 1 *mtp*-complement 1	Neat	Lawn	Lawn	Lawn	-	-	1.2×10^6
	10^{-1}	>200	>200	>200	-	-	
	10^{-2}	127	114	105	115.33	1.2×10^6	
	10^{-3}	19	10	11	-	-	
Biological assay 1 *mtp*-complement 2	Neat	Lawn	Lawn	Lawn	-	-	
	10^{-1}	>200	>200	>200	-	-	
	10^{-2}	118	124	117	119.67	1.2×10^6	
	10^{-3}	18	14	12	-	-	

WT, wildtype *M. tuberculosis* V9124; Δ*mtp, M. tuberculosis mtp*-deletion mutant; *mtp*-complement; CFU, colony forming unit.

$$CFU/mL = \frac{\text{(average number of colonies} \times \text{dilution factor)}}{\text{volume plated out in mL (0.01)}}$$

Table S4. Colony forming units (CFUs) of the biological assay 2 of the THP-1 macrophage infections.

Strain	Dilution	Replicate 1	Replicate 2	Replicate 3	Average	CFU/mL	Average CFU/mL
Biological assay 2 WT 1	Neat	Lawn	Lawn	Lawn	-	-	1.2×10^6
	10^{-1}	>200	>200	>200	-	-	
	10^{-2}	116	115	120	117.00	1.2×10^6	
	10^{-3}	11	12	12	-	-	
Biological assay 2 WT 2	Neat	Lawn	Lawn	Lawn	-	-	
	10^{-1}	>200	>200	>200	-	-	
	10^{-2}	130	103	115	116.00	1.2×10^6	
	10^{-3}	11	16	8	-	-	
Biological assay 2 Δ*mtp* 1	Neat	Lawn	Lawn	Lawn	-	-	1.0×10^6
	10^{-1}	>200	>200	>200	-	-	
	10^{-2}	97	98	104	99.33	9.9×10^5	
	10^{-3}	3	4	9	-	-	
Biological assay 2 Δ*mtp* 2	Neat	Lawn	Lawn	Lawn	-	-	
	10^{-1}	>200	>200	>200	-	-	
	10^{-2}	99	114	119	110.67	1.1×10^6	
	10^{-3}	9	16	13	-	-	
Biological assay 2 *mtp*-complement 1	Neat	Lawn	Lawn	Lawn	-	-	1.3×10^6
	10^{-1}	>200	>200	>200	-	-	
	10^{-2}	127	128	132	129.00	1.3×10^6	
	10^{-3}	9	10	14	-	-	
Biological assay 2 *mtp*-complement 2	Neat	Lawn	Lawn	Lawn	-	-	
	10^{-1}	>200	>200	>200	-	-	
	10^{-2}	128	124	136	129.33	1.3×10^6	
	10^{-3}	19	11	12	-	-	

WT, wildtype *M. tuberculosis* V9124; Δ*mtp, M. tuberculosis mtp*-deletion mutant; *mtp*-complement; CFU, colony forming unit.

.

$$CFU/mL = \frac{(\text{average number of colonies} \times \text{dilution factor})}{\text{volume plated out in mL } (0.01)}$$

Table S5. Colony forming units (CFUs) of the biological assay 3 of the THP-1 macrophage infections.

Strain	Dilution	Replicate 1	Replicate 2	Replicate 3	Average	CFU/mL	Average CFU/mL
Biological assay 3 WT 1	Neat	Lawn	Lawn	Lawn	-	-	1.2×10^6
	10^{-1}	>200	>200	>200	-	-	
	10^{-2}	118	126	125	123.00	1.2×10^6	
	10^{-3}	9	10	15	-	-	
Biological assay 3 WT 2	Neat	Lawn	Lawn	Lawn	-	-	
	10^{-1}	>200	>200	>200	-	-	
	10^{-2}	128	111	121	120.00	1.2×10^6	
	10^{-3}	7	11	19	-	-	
Biological assay 3 Δ*mtp* 1	Neat	Lawn	Lawn	Lawn	-	-	1.0×10^6
	10^{-1}	>200	>200	>200	-	-	
	10^{-2}	96	101	92	96.33	9.6×10^5	
	10^{-3}	2	16	8	-	-	
Biological assay 3 Δ*mtp* 2	Neat	Lawn	Lawn	Lawn	-	-	
	10^{-1}	>200	>200	>200	-	-	
	10^{-2}	99	117	103	106.33	1.1×10^6	
	10^{-3}	9	14	12	-	-	
Biological assay 3 *mtp*-complement 1	Neat	Lawn	Lawn	Lawn	-	-	1.3×10^6
	10^{-1}	>200	>200	>200	-	-	
	10^{-2}	130	126	132	129.33	1.3×10^6	
	10^{-3}	14	10	12	-	-	
Biological assay 3 *mtp*-complement 2	Neat	Lawn	Lawn	Lawn	-	-	
	10^{-1}	>200	>200	>200	-	-	
	10^{-2}	124	120	117	120.33	1.2×10^6	
	10^{-3}	19	7	14	-	-	

WT, wildtype *M. tuberculosis* V9124; Δ*mtp, M. tuberculosis mtp*-deletion mutant; *mtp*-complement; CFU, colony forming unit.

$$CFU/mL = \frac{(\text{average number of colonies} \times \text{dilution factor})}{\text{volume plated out in mL } (0.01)}$$

Table S6. Nanodrop readings of cultured bacterial RNA for three biological assays in triplicate for the three strains of *M. tuberculosis*.

Biological assay	Sample	Concentration (ng/µL)	$A_{260/280}$	$A_{260/230}$
1	WT 1	2047.0	1.69	1.57
	WT 2	1631.8	1.61	1.46
	*WT 3	1927.4	1.72	1.61
	Δ*mtp* 1	1194.8	1.72	1.62
	*Δ*mtp* 2	1423.4	1.80	1.72
	Δ*mtp 3*	906.1	1.73	1.66
	**mtp*-complement 1	2708.1	1.86	1.79
	mtp-complement 2	2210.0	1.84	1.81
	mtp-complement 3	1633.1	1.73	1.64
2	WT 1	1352.5	1.59	1.40
	*WT 2	1402.8	1.54	1.35
	WT 3	1406.5	1.63	1.49
	Δ*mtp* 1	1141.2	1.53	1.35
	*Δ*mtp* 2	1191.2	1.44	1.25
	Δ*mtp 3*	1237.4	1.41	1.18
	mtp-complement 1	3885.2	1.75	1.65
	mtp-complement 2	2976.7	1.66	1.52
	**mtp*-complement 3	2381.4	1.46	1.25
3	WT 1	1112.6	1.60	1.47
	*WT 2	1078.9	1.50	1.33
	WT 3	1326.0	1.44	1.24
	*Δ*mtp* 1	1335.6	1.64	1.51
	Δ*mtp* 2	1073.8	1.62	1.49
	Δ*mtp 3*	906.1	1.44	1.25
	mtp-complement 1	1550.1	1.73	1.63
	**mtp*-complement 2	1971.3	1.77	1.72
	mtp-complement 3	2001.1	1.83	1.81

WT, wildtype *M. tuberculosis* V9124; Δ*mtp*, *M. tuberculosis mtp*-deletion mutant; *mtp*-complement, *M. tuberculosis mtp*-complement; *, samples selected for DNase treatment and cDNA synthesis.

a)

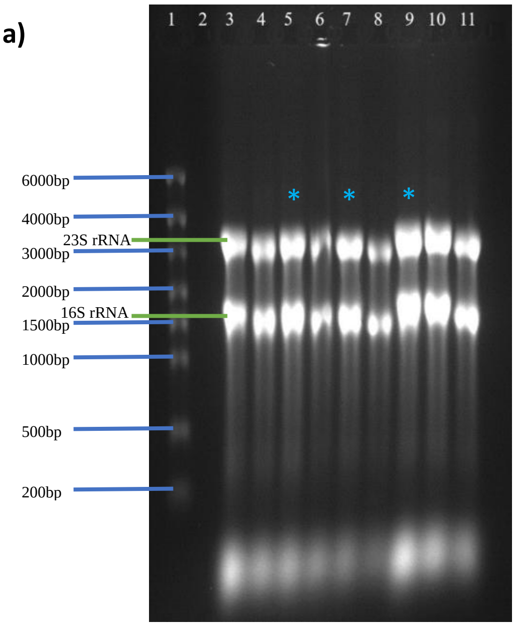

Biological assay 1 key:	
Lane 1	RiboRuler High Range RNA Ladder
Lane 3 - 5	WT replicates
Lane 6 - 8	Δ*mtp* replicates
Lane 9 - 11	*mtp*-complement replicates

b)

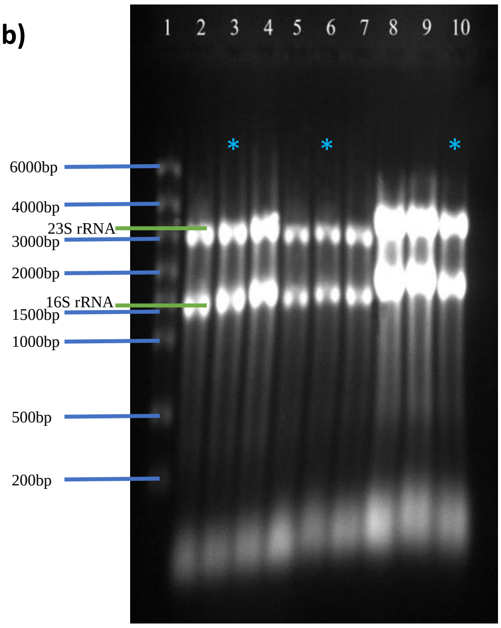

Biological assay 2 key:	
Lane 1	RiboRuler High Range RNA Ladder
Lane 2 - 4	WT replicates
Lane 5 - 7	Δ*mtp* replicates
Lane 8 - 10	*mtp*-complement replicates

c)

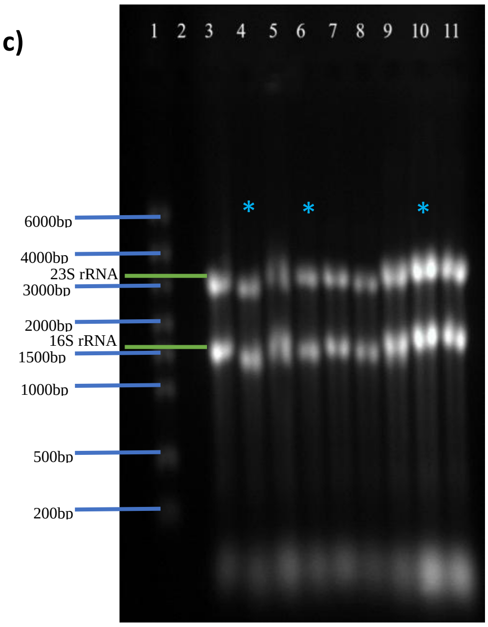

Biological assay 3 key:	
Lane 1	RiboRuler High Range RNA Ladder
Lane 3 - 5	WT replicates
Lane 6 - 8	Δ*mtp* replicates
Lane 9 - 11	*mtp*-complement replicates

Figure S1. Denaturing 3-(N-morpholino)propanesulfonic acid (MOPS) gel of all pure cultured bacterial samples from the three biological assays. All three MOPS gels were made with 1.5% agarose and run at 60 V for two hours. (a) Biological assay 1, with the molecular weight marker (RiboRuler High Range RNA Ladder) in the first lane, followed by the replicates of each strain of *M. tuberculosis* (WT, Δ*mtp*, *mtp*-complement) in sequential order. (b) Biological assay 2, with the molecular weight marker in the first lane, followed by the replicates of each strain of *M. tuberculosis* (WT, Δ*mtp*, *mtp*-complement) in sequential order. (c) Biological assay 3, with the molecular weight marker in the first lane, followed by the replicates of each strain of *M. tuberculosis* (WT, Δ*mtp*, *mtp*-complement) in sequential order. All samples showed two bands (23S ribosomal RNA, approximately 3000 bp and 16S ribosomal RNA, approximately 1500 bp). *, samples selected for DNase treatment and cDNA conversion.

Table S7. Intracellular bacterial RNA concentrations and purity ratios for all three biological assays:

Biological assay	Sample	RNA concentration (ng/uL)	$A_{260/280}$	$A_{260/230}$
1	*WT 1	2273.5	1.88	2.17
	WT 2	2105.3	1.85	2.14
	*Δ*mtp* 1	2213.2	1.90	2.15
	Δ*mtp* 2	2202.7	1.89	2.18
	**mtp*-complement 1	3566.9	1.95	2.17
	mtp-complement 2	3485.9	1.80	2.10
2	*WT 1	2081.2	1.95	2.19
	WT 2	2071.2	1.90	2.16
	*Δ*mtp* 1	2224.5	1.95	2.17
	Δ*mtp* 2	2201.8	1.90	2.13
	**mtp*-complement 1	2344.0	1.95	2.11
	mtp-complement 2	2332.2	1.93	2.10
3	*WT 1	2316.1	1.92	2.09
	WT 2	2349.9	1.92	2.12
	*Δ*mtp* 1	2921.5	2.02	2.20
	Δ*mtp* 2	2891.2	2.02	2.21
	**mtp*-complement 1	1343.5	2.00	2.18
	mtp-complement 2	1363.1	1.99	2.20

WT, wildtype *M. tuberculosis* V9124; Δ*mtp*, *M. tuberculosis mtp*-deletion mutant; *mtp*-complement; *, samples selected for DNase treatment and cDNA synthesis.

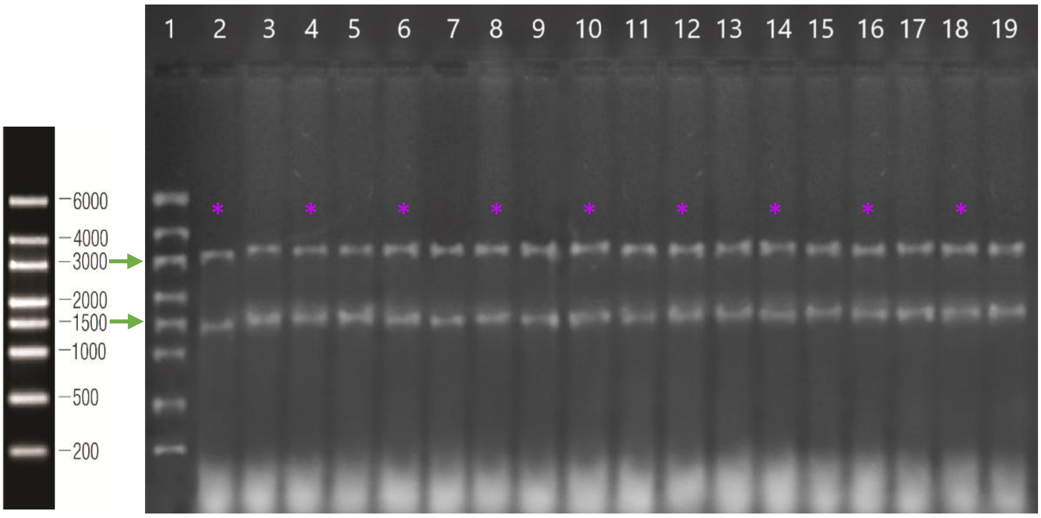

Figure S2. Denaturing MOPS gel of all strains in duplicate for the three biological assays of intracellular bacteria. The MOPS gel was comprised 1.5% agarose and was run at 70 V for 3 hours. RiboRuler high range RNA ladder was used as the molecular weight marker in lane 1. Two bands were expected around 3000 bp (23s ribosomal RNA) and 1500 bp (16s ribosomal RNA). Biological assay 1 is shown in lanes 2-7; biological assay 2 is shown in lanes 8-13 and biological assay 3 is shown in lanes 14-19. Lanes 2 and 3, 8 and 9, and 14 and 15 have RNA samples for WT duplicate for the respective biological assay; lanes 4 and 5, 10 and 11, and 16 and 17 have RNA samples for Δ*mtp* duplicate for the respective biological assay; and lanes 6 and 7, 12 and 13, and 18 and 19 have RNA samples for *mtp*-complement duplicate for the respective biological assay. *, samples selected for DNase treatment and cDNA conversion.

Table S8. Concentration and purities of cDNA from the selected cultured bacterial samples after DNase treatment and cDNA synthesis for all three biological assays used for 16s rRNA PCR confirmation and RT-qPCR.

Sample	cDNA concentration (ng/ µL)	$A_{260/280}$	$A_{260/230}$
Biological assay 1: WT 3	2577.1	1.78	2.21
Biological assay 2: WT 2	2634.6	1.77	2.19
Biological assay 3: WT 2	1927.6	1.80	2.22
Biological assay 1: Δ*mtp* 2	2652.4	1.78	2.22
Biological assay 2: Δ*mtp* 2	2726.8	1.76	2.18
Biological assay 3: Δ*mtp* 1	1796.8	1.81	2.24
Biological assay 1: *mtp*-complement 1	2649.3	1.77	2.21
Biological assay 2: *mtp*-complement 3	2672.4	1.77	2.18
Biological assay 3: *mtp*-complement 2	1637.7	1.81	2.26

WT, wildtype *M. tuberculosis* V9124; Δ*mtp, M. tuberculosis mtp*-deletion mutant; *mtp*-complement.

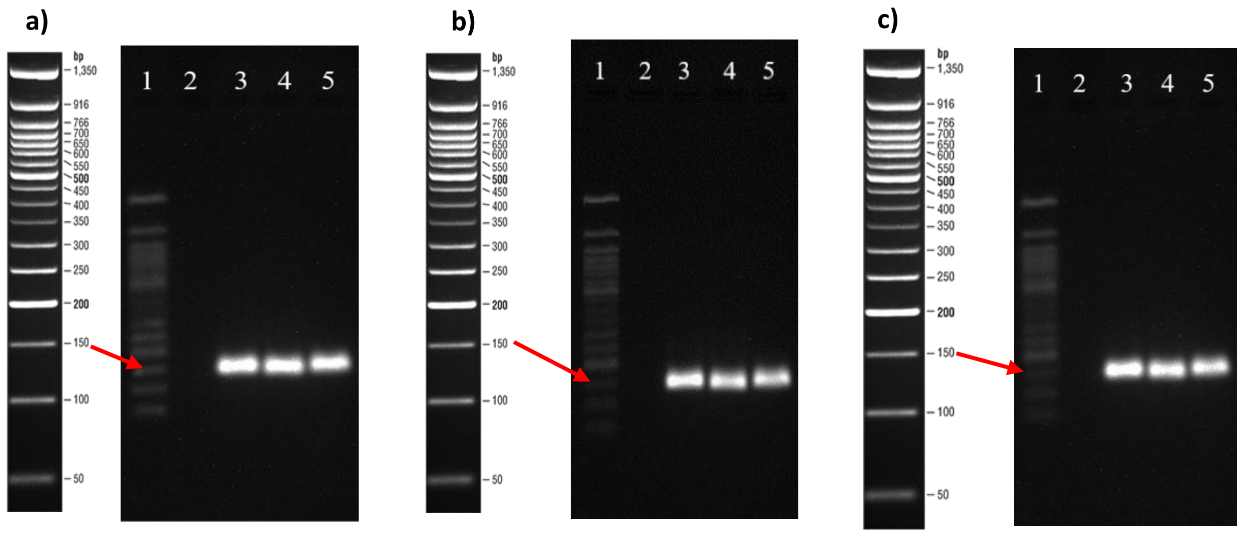

Figure S3. 16S ribosomal RNA PCR confirmation gels for cultured bacteria. All samples were run on 1.5% agarose gels for 90 minutes at 60 V. Each biological assay was run on separate gels. The expected product size of the 16S rRNA target was 151 bp, which was present for each strain in each biological assay. The molecular weight marker (Quick-Load Purple 50 bp DNA ladder) is in lane 1 of all gels. Lane 3 is the WT, lane 4 is the Δ*mtp*, and lane 5 is the *mtp*-complement for (a) biological assay 1, (b) biological assay 2, and (c) biological assay 3, respectively.

Table S9. Concentration and purities of cDNA from the selected intracellular bacterial samples after DNase treatment and cDNA synthesis for all three biological assays used for 16s rRNA PCR confirmation and RT-qPCR:

**Sample**	**cDNA concentration (ng/ µL)**	**$A_{260/280}$**	**$A_{260/230}$**
Biological assay 1: WT 1	2348.5	1.83	2.30
Biological assay 2: WT 1	2136.1	1.83	2.30
Biological assay 3: WT 1	2423.8	1.82	2.28
Biological assay 1: Δ*mtp* 1	1642.4	1.84	2.32
Biological assay 2: Δ*mtp* 1	2261.0	1.83	2.29
Biological assay 3: Δ*mtp* 1	2039.2	1.83	2.30
Biological assay 1: *mtp*-complement 1	2278.8	1.82	2.28
Biological assay 2: *mtp*-complement 1	2044.4	1.83	2.30
Biological assay 3: *mtp*-complement 1	2259.7	1.83	2.29

WT, wildtype *M. tuberculosis* V9124; Δ*mtp, M. tuberculosis mtp*-deletion mutant; *mtp*-complement.

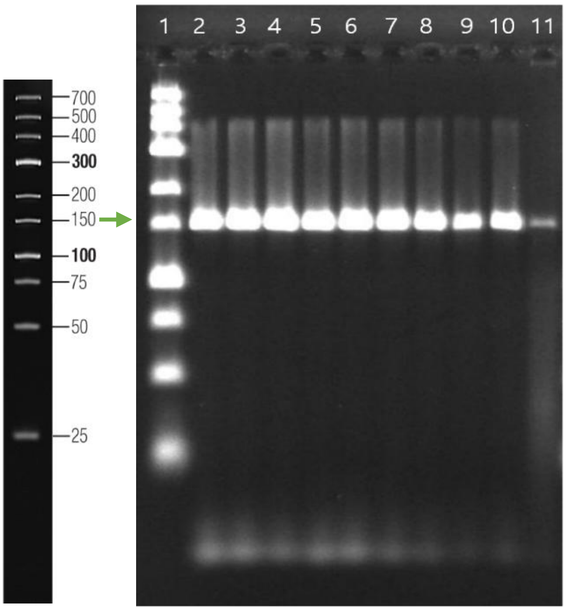

Figure S4. 16S ribosomal RNA confirmation of intracellular bacteria from THP-1 macrophage infection. The 3% agarose gel was run at 90 V for 75 minutes. GeneRuler low range DNA ladder was used as the molecular weight marker in lane 1. The expected product size is 151 bp. WT, Δ*mtp* and *mtp*-complement form biological assay 1 is depicted in lanes 2, 3 and 4, respectively. WT, Δ*mtp* and *mtp*-complement form biological assay 2 is depicted in lanes 5, 6 and 7, respectively. WT, Δ*mtp* and *mtp*-complement form biological assay 3 is depicted in lanes 8, 9 and 10, respectively. A positive bacterial control was included in lane 11.

Table S10. Primer sequences, product sizes and annealing temperatures.

Gene name	Forward Primer Sequence (5'-3')	Reverse Primer Sequence (5'-3')	Product size (bp)	Annealing temperature (°C)
Rv3723/lucA	ccggtggtattcactttgct	cgacaggtagaacccgaaga	234	61
Rv2799	agggggtgtcgtgttcacc	gacgtcgaacgggatgtctc	235	60
Rv0966c	ctatgcggcaaccacctacc	gtcagcttcttcggcacgtt	171	61
Rv0200/omamB	tggtggtgttcgatgtcctg	acgacgcaacagccagaagt	158	61
Rv0172/mce1D	ggcaagggtaagcaaatcaa	ggtcaacctgtcggtgaact	189	59
Rv0655/mceG	tgacatcgtcatggagaagc	gatcagctggctcaggtagg	196	60
Rv0860/fadB	gtcaaccaacgtgatgaacg	ggcttacccaatgtctccaa	237	60
Rv3546/fadA5	catctgattgccgggttgat	actggttcggcaggtcgata	154	60
Rv0694/lldD1	ggggtactgtgatgggattg	tcatctcttcggggatcttg	228	59
Rv1872c/lldD2	acccctgctccagttcaaca	gaaactcgatgtcgcggaac	194	60
Rv0211/pckA	ccgagaagcacaagaactcc	acagaacggcaccacataca	206	61
16S rRNA *MTB000019/rrs*	ggcgtgcttaacacatgcaa	catgcatcccgtggtcctat	151	60

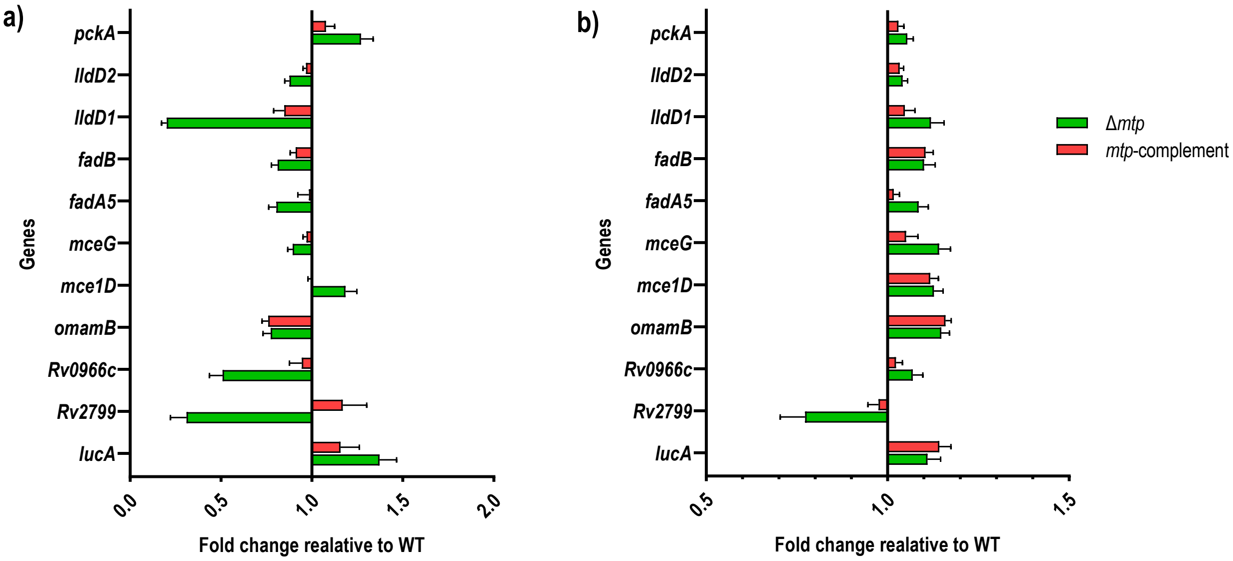

Figure S5. Fold changes of gene expression levels relative to the WT groups. The absolute quantification of gene expression was determined through RT-qPCR of the (a) cultured bacterial strains, and (b) intracellular bacterial strains isolated from THP-1 macrophages (WT V9124; Δ*mtp*-deletion mutant; and *mtp*-complemented strain). Three biological assays and four technical repeats were conducted for each of the 11 selected genes (*lucA, Rv2799, Rv0966c, omamB, mce1D, mceG, fadA5, fadB, lldD1, lldD2,* and *pckA*). Gene expression levels were normalized to the housekeeping 16S rRNA for each gene of interest. Fold change is relative to the respective WT group for (a) and (b) and were represented by standard error mean (SEM) bars. A fold change of 1 denotes that gene expression levels were identical to the WT group. Fold changes lower than 1 denoted that the gene expression levels were lower than the WT group, and fold changes higher than 1 denoted that the gene expression levels were higher than the WT group.
